# Operationalising targeted next-generation sequencing for routine diagnosis of drug-resistant TB

**DOI:** 10.5588/pha.22.0041

**Published:** 2023-06-21

**Authors:** A. Iyer, Z. Ndlovu, J. Sharma, H. Mansoor, M. Bharati, S. Kolan, M. Morales, M. Das, P. Issakidis, G. Ferlazzo, N. Hirani, A. Joshi, P. Tipre, N. Sutar, K. England

**Affiliations:** 1 Médecins Sans Frontières (MSF), Mumbai, India; 2 MSF, Southern African Medical Unit, Cape Town, South Africa; 3 Division of Epidemiology and Biostatistics, Department of Global Health, Faculty of Medicine and Health Sciences, Stellenbosch University, Cape Town, South Africa; 4 Department of Mycobacteriology, Sir JJ Group of Hospitals, Mumbai, India; 5 National Tuberculosis Elimination Programme, Mumbai, India; 6 Independent Consultant, Honolulu, HI, USA

**Keywords:** tNGS, Deeplex-MycTB, resistotyping, MDR-TB

## Abstract

**BACKGROUND:**

Phenotypic drug susceptibility testing (pDST) for *Mycobacterium tuberculosis* can take up to 8 weeks, while conventional molecular tests identify a limited set of resistance mutations. Targeted next-generation sequencing (tNGS) offers rapid results for predicting comprehensive drug resistance, and this study sought to explore its operational feasibility within a public health laboratory in Mumbai, India.

**METHODS:**

Pulmonary samples from consenting patients testing Xpert MTB-positive were tested for drug resistance by conventional methods and using tNGS. Laboratory operational and logistical implementation experiences from study team members are shared below.

**RESULTS:**

Of the total number of patients tested, 70% (113/161) had no history of previous TB or treatment; however, 88.2% (*n* = 142) had rifampicin-resistant/multidrug-resistant TB (RR/MDR-TB). There was a high concordance between resistance predictions of tNGS and pDST for most drugs, with tNGS more accurately identifying resistance overall. tNGS was integrated and adapted into the laboratory workflow; however, batching samples caused significantly longer result turnaround time, fastest at 24 days. Manual DNA extraction caused inefficiencies; thus protocol optimisations were performed. Technical expertise was required for analysis of uncharacterised mutations and interpretation of report templates. tNGS cost per sample was US$230, while for pDST this was US$119.

**CONCLUSIONS:**

Implementation of tNGS is feasible in reference laboratories. It can rapidly identify drug resistance and should be considered as a potential alternative to pDST.

## Abstract (fr)

**CONTEXTE:**

Les tests phénotypiques de sensibilité aux médicaments (pDST) pour *Mycobacterium tuberculosis* peuvent prendre jusqu’à 8 semaines, tandis que les tests moléculaires conventionnels identifient un ensemble limité de mutations de résistance. Le séquençage ciblé de la prochaine génération (tNGS) offre des résultats rapides pour prédire la résistance globale aux médicaments, et cette étude avait pour objectif d’explorer sa faisabilité opérationnelle au sein d’un laboratoire de santé publique à Mumbai, en Inde.

**MÉTHODES:**

Des échantillons pulmonaires de patients consentants testés positifs au Xpert MTB ont été testés pour la résistance aux médicaments par des méthodes conventionnelles et en utilisant le tNGS. Les expériences des membres de l’équipe de l’étude en matière de fonctionnement du laboratoire et de mise en œuvre logistique sont présentées ci-dessous.

**RÉSULTATS:**

Sur le nombre total de patients testés, 70% (113/161) n’avaient pas d’antécédents de TB ou de traitement ; cependant, 88,2% (*n* = 142) présentaient une TB résistante à la rifampicine/multirésistante aux médicaments (RR/MDR-TB). La concordance entre les prédictions de résistance de la tNGS et de la pDST était élevée pour la plupart des médicaments, la tNGS identifiant globalement la résistance avec plus de précision. La tNGS a été intégrée et adaptée au flux de travail du laboratoire ; toutefois, la mise en lots des échantillons a entraîné un délai d’obtention des résultats beaucoup plus long, le plus rapide étant de 24 jours. L’extraction manuelle de l’ADN a été source d’inefficacité ; le protocole a donc été optimisé. L’analyse des mutations non caractérisées et l’interprétation des modèles de rapport ont nécessité une expertise technique. Le coût du tNGS par échantillon s’élevait à US$230, contre US$119 pour le pDST.

**CONCLUSIONS:**

La mise en œuvre de la tNGS est possible dans les laboratoires de référence. Elle permet d’identifier rapidement la résistance aux médicaments et devrait être considérée comme une alternative potentielle à la pDST.

In 2020, there was an estimated 465,000 incident cases of rifampicin-resistant TB (RR-TB), with 78% having multidrug-resistant TB (MDR-TB).^1^ India had the largest global burden of MDR-TB (27%), followed by China (14%). Less than 30% of the estimated incident DR-TB was diagnosed and less than 50% of all incident cases accessed treatment.^1^ To control the expansion of DR-TB, rapid and comprehensive diagnosis is needed.

Phenotypic drug susceptibility testing (pDST) remains the gold standard for the identification of resistance to currently used anti-TB drugs, but is known to be unreliable for most drugs taking up to 8 weeks for results.^2^ Newer molecular methods provide rapid detection for *Mycobacterium tuberculosis* (MTB) but identify resistance to only a few anti-TB drugs.^3^ The emergence of MTB strains with complex drug resistance profiles necessitates the need for rapid comprehensive resistance determination to guide patient treatment.^4^ Advanced molecular technologies such as next-generation sequencing (NGS) have the potential for rapidly diagnosing DR-TB by providing detailed sequence information for multiple gene regions (or whole genome), overcoming challenges associated with pDST and limitations with currently used molecular tests.

In early pilot testing, high-throughput benchtop NGS technology demonstrated advantages that suggests it could complement or replace pDST for the diagnosis of DR-TB.^5,6^ However, the uptake of NGS for DR-TB diagnosis has been hindered by concerns regarding their costs, integration into existing laboratory workflows, and required technical skills.

This paper presents lessons from a pilot implementation, which sought to assess the operational feasibility of targeted NGS (tNGS) in a public health reference laboratory as a routine diagnostic test for predicting drug resistance among patients in a high DR-TB ­burden setting. Operational feasibility is reported in ­relation to sample management, integration into laboratory workflows, data reporting and turnaround time (TAT) for results, including costing. Detailed performance and clinical utility from this study is described by Mansoor et al.^7^

## METHODS

### Study setting and programme description

Since 2016, Médecins Sans Frontières (MSF) partnered with National Tuberculosis Elimination Programme (NTEP) of India to provide support for DR-TB services at M-East Ward (MEW), one of the highest DR-TB burden wards in Mumbai.

A sequencing platform (Illumina MiSeq, San Diego, CA, USA) was previously donated to Sir Jamshedjee Jeejebhoy (JJ) Hospital’s Microbiology Laboratory in 2016 by the Foundation for Innovative New Diagnostics (FIND; Geneva, Switzerland). The JJ Hospital Laboratory is an accredited biosafety level 3 (BSL3) National Reference Laboratory (NRL) for TB/DR-TB testing. MSF provided tNGS testing reagents, consumables, and other resources, and supported training by GenoScreen (Lille, France) associates to initiate this pilot study.

### Study design and participants

**FIGURE 1:**
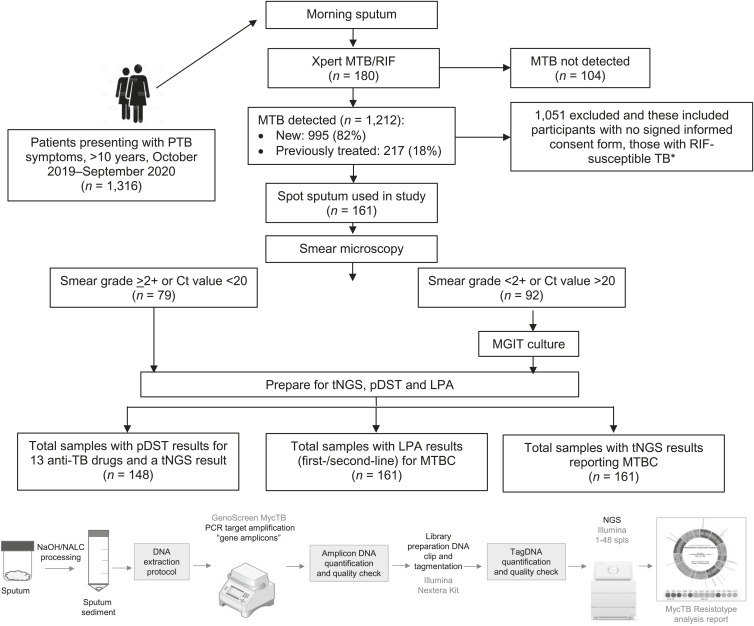
Specimen and laboratory workflow. *Shift in selection of RIF resistance over susceptible samples occurred during the study to better understand mutations currently in circulation for second-line drugs. MTB = *M. tuberculosis*; PTB = pulmonary TB; RIF = rifampicin; Ct = cycle threshold; MGIT = Mycobacteria Growth Indicator Tube; tNGS = targeted next-generation sequencing; pDST = phenotypic drug susceptibility testing; LPA = line-probe assay; MTBC = *M. tuberculosis* complex; NaOH = sodium hydroxide; NALC = *N*-acetyl-L-cysteine; PCR = polymerase chain reaction.

Pulmonary samples testing MTB-positive on Xpert^®^ MTB/RIF (Cepheid, Sunnyvale, CA, USA) regardless of RR-TB were collected from consenting participants from Shatabdi Hospital at MEW and private referrals through October 2019 to September 2020. Patients presenting with extrapulmonary TB (with or without co-existent pulmonary TB) or those younger than 10 years of age were excluded. Samples were transported to the JJ Hospital Laboratory for testing (Figure 1) and the following tests were performed: tNGS, first-/second-line line-probe assay (LPA) and MGIT (Mycobacteria Growth Indicator Tube™; BD, Franklin Lakes, NJ, USA) pDST for 13 anti-TB drugs: isoniazid (INH), rifampicin (RIF), ethambutol (EMB), pyrazinamide (PZA), streptomycin (SM), and fluoroquinolones (FQs) [levofloxacin (LVX), moxifloxacin (MFX)], kanamycin (KM), amikacin (AMK), capreomycin (CPM), ethionamide (ETO), linezolid (LZD) and clofazimine (CFZ); critical concentrations as recommended in the NTEP guidelines were used (Table 1). Bedaquiline (BDQ) pDST was not performed as it was not yet routinely implemented.

**TABLE 1:** GenoScreen MycTB tNGS targets amplified for tNGS by drug compound and critical concentration used in pDST

Drug	Gene targets	Critical concentration μg/mL
INH	*kat*G*, inh*A*, fabG1, ahp*C	0.1
RIF	*rpo*B	1.0
EMB	*emb*B	5.0
PZA	*pnc*A	100.0
SM	*rrs, rps*L*, gid*B	1.0
FQ	*gyr*A*, gyr*B	LVX (1.0), MFX^L ^(0.25), MFX^H^ (1.0)
KM	*eis* *, rrs*	2.5
AMK	*rrs*	1.0
CPM	*rrs, tly*A	2.5
ETH	*eth*A*, fabG1, inh*A	5.0
LZD	*rplC* *, rrl*	1.0
CFZ	*rv0678*	1.0

tNGS = targeted next-generation sequencing; pDST = phenotypic drug susceptibility testing; INH = isoniazid; RIF = rifampicin; EMB = ethambutol; PZA = pyrazinamide; SM = streptomycin; FQ = fluoroquinolone; LVX = levofloxacin; MFX^L^ = low-dose moxifloxacin; MFX^H^ = high-dose moxifloxacin; KM = kanamycin; AMK = amikacin; CPM = capreomycin; ETH = ethionamide; LZD = linezolid; CFZ = clofazimine.

### Laboratory procedures

Two laboratory technicians (microbiologists) with more than 5 years TB diagnostic experience, had a 7-day on-site training as master users of the Illumina MiSeq for tNGS by the GenoScreen and Illumina technical teams.

#### Study specimens

Morning samples with a cycle threshold (Ct) value of less than 20 on Xpert, or those with a positive direct smear grade in spot samples, were prioritised for direct sputum tNGS analysis over culture-enriched samples. Sputum samples were decontaminated and processed according standard NaOH/NALC (sodium hydroxide/*N*-acetyl-L-cysteine) protocols^8^ and sedimented cells were split for testing as follows: 500 μL for LPA testing, 500 μL for MGIT culture and 500 μL for gDNA extraction/tNGS. The remaining was stored for retesting as needed.

#### Rapid molecular testing

Xpert MTB/RIF and GenoType MTBDR*plus* and GenoType MTBDR*sl* (Bruker-Hain Lifesciences, Nehren, Germany) were used to detect resistance to first-line drugs (RIF, INH) and second-line drugs: FQ plus injectables (KM, AMK, CPM).

#### Phenotypic drug susceptibility testing

BACTEC™ MGIT™ 960 (Becton Dickinson, Sparks, MD, USA) was used for pDST following the 2018 WHO critical concentrations for drugs.^9^

#### DNA extraction, library preparation, sequencing and data analysis

DNA extraction, target amplification and tNGS were performed as described in the Deeplex-MycTB kit (GenoScreen). Deep sequencing uses a 24-plexed amplicon mix for simultaneous mycobacterial species identification, MTB genotyping and resistance prediction covering 18 gene targets to commonly used anti-TB drugs.^10^

Amplicons were purified using NucleoMag Magnetic Beads (Macherey Nagel, Duren, Germany) and quantified using Qubit dsDNA BR assay (Life Technologies, Paisley, UK). Paired-end libraries (150 base pair) were prepared using Nextera XT DNA Sample Preparation kits (Illumina Inc; San Diego, CA, USA) and sequenced (depth 100x) on an Illumina MiSeq platform. To determine drug resistance, we utilized GenoScreen v7.7.9, a cloud-based analytical platform that incorporates published reference datasets of genetic variants associated with drug resistance.^11,12^ In cases where a clear resistance determination was not obtained, we engaged a TB sequence data specialist to analyse post-sequencing data and assess coverage and depth for the samples.

To inform the operational feasibility of tNGS, sample management (including sputum smear grades, DNA extraction and amplification), sequencing and its integration into laboratory workflows (wet and dry laboratory work spaces), data analysis and costing, as well as laboratory efficiencies were explored. The study team (laboratory technicians, laboratory management, laboratory advisors, clinicians, consultants and TB sequence specialists) held regular meetings to share and document operational and logistical experiences in the implementation of tNGS, which are shared below.

The study received ethics approval from the Institutional Review Board, Grant Government Medical College, Mumbai, India; and Sir JJ Groups of Hospitals, Mumbai, India.

## RESULTS

### Characteristics of study participants

The overall median age in the study was 24 years (interquartile range [IQR] 20–40); 57% (92/161) were female (Table 2). Of the 161 resistant patients, 70% (113/161) had no history of previous TB treatment; however, 88.2% (142/161) had RR/MDR-TB with 58.5% having FQ resistance, i.e., pre-extensively drug-resistant TB (pre-XDR-TB) and 9.2% with additional resistance to either LZD or BDQ (XDR-TB) as noted by Mansoor et al.^7^

**TABLE 2:** Demographic and clinical characteristics of patients enrolled (*n = *161)

Variable	*n* (%)
Sex	
Male	69 (43)
Female	92 (57)
Age category, years	
12–19	47 (29)
20–29	50 (31)
30–39	31 (19)
40–49	11 (7)
⩾50	22 (14)
Age, years, median [IQR]	24 [20–40]
Healthcare institution	
Patients from public sector	108 (67)
Patients from private practitioners	53 (33)
Previous TB	
Yes	40 (25)
No	113 (70)
Unknown	8 (5)
Culture at baseline	
Positive	148 (92)
Negative	13 (8)
Resistance profile	
Susceptible to all TB drugs	15 (9.3)
INH-monoresistant TB	1 (0.6)
FQ-monoresistant TB	2 (1.2)
Other	1 (0.6)
RR/MDR-TB	142 (88.2)
WHO 2020 definitions	
Pre-XDR-TB (FQ or injectable)	74 (52.1)
XDR-TB (FQ and injectable)	24 (16.9)
WHO 2021 definitions^13^	
Pre-XDR-TB (FQ)	83 (58.5)
XDR-TB (FQ + LZD or BDQ*)	13 (9.2)

*Samples with CFZ (*rv0678*) cross resistance, BDQ DST was not done.

IQR = interquartile range; INH = isoniazid; FQ = fluoroquinolone; RR/MDR-TB = rifampicin-resistant/multidrug-resistant TB; XDR-TB = extensively drug-resistant TB; LZD = linezolid; BDQ = bedaquiline.

### Diagnostic performance comparison: tNGS and conventional methods

**FIGURE 2:**
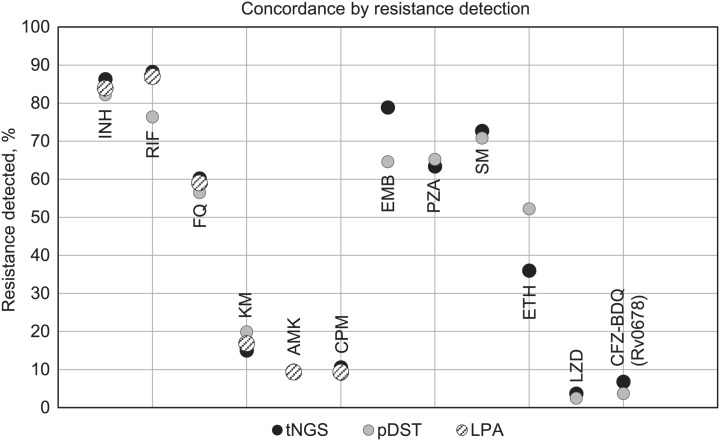
Comparison of tNGS, pDST and LPA in detection of anti-TB drug resistance. INH = isoniazid; RIF = rifampicin; FQ = fluoroquinolone; KM = kanamycin; AMK = amikacin; CPM = capreomycin; EMB = ethambutol; PZA = pyrazinamide; SM = streptomycin; ETH = ethionamide; LZD = linezolid; CFZ = clofazimine; BDQ = bedaquiline; tNGS = targeted next-generation sequencing; pDST = phenotypic drug susceptibility testing; LPA = line-probe assay.

tNGS predicted more resistance over pDST for most drugs, with the exception of resistance to EMB and ETH (Figure 2). Agreement between molecular methods was high, while discrepancies between pDST and tNGS was noted for low-level borderline resistance mutations known to pose challenges to pDST. Results on performance and clinical utility of tNGS from this study are described by Mansoor et al.^7^

### Operational feasibility of tNGS

In Supplementary Data 1, we provide an outline of the overall phased strategy that defines the elements for preparation, implementation and operationalisation of tNGS for routine clinical diagnosis within a laboratory setting. Throughout the course of implementation, various challenges were identified that would require mitigation measures to operationalise testing for routine use (Supplementary Data 2).

#### Sputum sample quality

A preliminary evaluation of sputum Ct value and/or smear grade was conducted as bacillary load was assumed to be crucial for quality sequence results. Therefore, only sputum samples with smear grades >2+ and or Ct <20 (79/161, 49.1%) were considered for direct sputum tNGS. All other samples were cultured prior to tNGS preparation.

#### tNGS workflow

In the ‘wet laboratory space’, manual DNA extraction and purification per batch of 45 samples took approximately 10 h (Deeplex protocol); polymerase chain reaction (PCR) amplification took 3 h. Library preparation (fragmentation, amplification and quantification) took between 5 and 7 h, with sequence to result ranging from 36 to 50 h. PCR amplification and library preparation steps required protocol modifications to optimise the quality and quantity of DNA. Initially, PCR amplification was performed using PCR plates rather than PCR tubes, leading to a loss of DNA through evaporation upon centrifugation (Supplementary Data 2). The use of low-binding nuclease-free PCR tubes resulted in higher DNA yield. Library preparation required a minimum of 2nM amplicon DNA, and samples below this threshold were discarded. Quality assessments were not performed for each sample, but randomly for 1–2 samples per batch; 9.3% (15/161) demonstrated poor quality (i.e., low coverage across at least one or more gene targets). Within the ‘dry laboratory space’, FASTQ data files were uploaded to GenoScreen’s web-based analysis software for analysis; however, due to poor internet connectivity, complete analyses ranged between 48 and 96 h. Overall, laboratory TAT per batch of 45 samples ranged from 6 to 7 days.

#### Data analysis and reporting

Result outputs were in the form of pdf reports (Supplementary Data 3) and were exported into MS Excel (Microsoft, Redmond, WA, USA) spreadsheets for details on genotyping, percentage variant population (heteroresistance), confidence indicators and target metrics (coverage breadth, depth and perfect variant levels) analysis. Post analysis was complex and required external support from GenoScreen technical support and an external consultant. Indeterminant genotypes reporting low coverage required further interrogation using the web-based application to call drug susceptibility. Uncharacterised variants with phenotypic resistance required added literature reviews to verify clinical relevance, as rapidly evolving resistance data were not sufficiently updated in databases. Furthermore, genotypic and phenotypic discrepancies required investigation for borderline (low-level) mutations or the presence of insertions and deletions known to pose issues in pDST. GenoScreen technical support provided guidance for the analyses and training for interpretation during the post-analysis process.

#### Result turnaround time

Due to many challenges, including batching of samples, median TAT for tNGS in culture-enriched samples (82/161, 50.9%) was 143 days, and the fastest TAT was 24 days among samples that did not require prior culture (Table 3).

**TABLE 3:** Test turnaround times for different testing methods

Test or process	Test turnaround time, days Median [IQR]
Xpert MTB/RIF	1 [1–3]
Transit to JJ Laboratory, Mumbai, India	2 [1–4]
FL LPA	14 [6–29]
SL LPA	25 [6–35]
Full panel DST	149 [55–200]
tNGS	143 [65–184]

IQR = interquartile range; FL = first-line; LPA = line-probe assay; SL = second-line; DST = drug susceptibility testing; tNGS = targeted next-generation sequencing.

#### Cost: start-up and routine

Equipment costs for start-up (with all new equipment) were estimated at US$160,000. The cost for reagent kits and consumables for DNA extraction, MycTB amplification, DNA quality and quantity checks, Nextera library preparation and sequencing was estimated per sample and per batch of 48 (45 clinical specimens and 3 controls) at US$230 per sample. Cost estimates per sample for other routine tests can be found in Table 4. Detailed costing and breakdowns for equipment, kits and consumables can be found in Supplementary Data 4; however, this excludes cost of labour.

**TABLE 4:** Estimates for cost per test (setting-specific)

Laboratory test	Cost (US$)
Xpert MTB/RIF	30.00
Bruker-Hain MTBDR*plus*	22.00
Bruker-Hain MTBDR*sl*	22.00
BD MGIT DST panel (13 drugs)	45.00
Total cost per patient sample	119.00

*Costs include local distributor (India) mark-ups and taxes.

MGIT = Mycobacteria Growth Indicator Tube; DST = drug susceptibility testing.

#### Procurement

In the preparatory phase of the study, equipment and reagents were procured in bulk. However, due to the COVID-19 pandemic which delayed sample collection, together with batching of samples; 3/6 (50%) test kits reached their expiration dates due to the short shelf life (especially the GenoScreen DNA and the Nextera library preparation kits, which had a 3–6-month shelf life). For this study, all expired reagents were replaced by the manufactures.

## DISCUSSION

Findings from this study suggest that NGS is implementable for comprehensive genotypic DST in high DR-TB burden settings. As this study is part of early attempts for piloting NGS, the work-streams still require further optimisation with improvements expected through increased experience.

In the present study, 79 (49.1%) direct sputum samples (>2+) gave quality results, while paucibacillary samples required culture preparation for tNGS. However, results demonstrated no correlation between bacillary load and tNGS performance. In addition, other studies have shown that all positive smear grades provide pan-target read depths of over 1000×, sufficient for predicting known resistance for all targeted drugs.^14,15^ However, injectable drugs exhibited low coverage and required further interrogation for key resistance mutations using the GenoScreen Web App for clarification.

DNA quality is important in achieving quality sequence results; however, due to extra cost implications, quality assessments using Agilent Bionalayzer (Agilent, Santa Clara, CA, USA) were performed for 1–2 samples per batch. Even with limited quality checks, only 9.3% demonstrated poor quality (low coverage across at least one or more gene targets (mainly *rrs*). To ensure consistency of DNA, performance of routine DNA quality checks, an automated nucleic acid extractor and bioanalyser kits for each sample should be considered. This experience demonstrated that manual genomic DNA extraction posed inefficiencies related to intensive and time-consuming procedures, increasing the risk for processing and human errors, including risks for cross-contamination.

Figure 2 shows that tNGS detected a greater degree of resistance than pDST for most drugs, indicating that tNGS may be a more reliable method for identifying drug resistance. However, it is worth noting that EMB and ETH, which are known to be unreliable in pDST, did not show a significant difference in resistance detection between the two methods.^16^ In comparison to the gold standard, MGIT pDST, concordance between resistance predictions for the first-line drugs in this study (exclusive of uncharacterised variants) ranged from 100% (RIF), 99% (INH), 100% (EMB) to 88% (PZA). The frequency of resistance for FQ and injectables ranged from 96% (FQ) to 63% and 88% (KM and AM, respectively).^7^ These findings compare with other studies that showed that Deeplex-MycTB is superior to molecular assays.^17–20^ The variant data in the study align with the WHO mutations catalogue of resistance-associated genetic variants for predicting clinically relevant resistance.^21^

The shortest TAT for tNGS results observed was 24 days. Delays from batching of samples and COVID-19 pandemic restrictions, which minimised patient access to services, contributed to the delays. However, other studies have reported TATs of 4–7 days, and this also depends on the rate of sample submissions, internet quality and staff experience.^15^

Molecular biologists or trained laboratory technicians with understanding of DR-TB mutations are required for conducting and interpreting NGS data. The time dedicated to fulfil NGS workflow training can take days to weeks, depending on the experience of staff. A rapid colour-coded resistotype report is generated per case, limiting the need for having a bioinformatician onsite. However, a high level of the competency for troubleshooting and interpreting discrepancies between genotypic and phenotypic results is essential. Study experiences highlight foreseen challenges in the interpretation and communication of NGS information in an easily understood format for clinicians. The current reporting template from GenoScreen software remains complex for direct reporting to clinicians. This underscores the need for standardised clinical reporting of genomic information to maximise its utility. Furthermore, training of both clinicians and laboratorians on the utility of tNGS, results interpretation and key challenges is fundamental, particularly as regards to the characterisation of novel mutations.

The running costs per sample from DNA extraction to the resulting sequence was US$230. There is limited data on routine tNGS cost per sample, as most studies do not collate the entire cost from DNA extraction, library preparation, sequencing and all associated reagents.^5,22^ Nonetheless, the overall patient cost may be less if treated earlier with an optimised regimen. In settings with low sampling numbers, use of test kits that allow smaller batches could improve speed and cost efficiency. Illumina NGS can be conducted on platforms with various throughputs: iSeq100 (*n* = 28), MiniSeq (*n* = 50), MiSeq (*n* = 96) and NextSeq (*n* = 384).^5^ Detailed descriptions of commercially available NGS technologies have been reported,^5,15^ and laboratories may select a platform that best meets their needs.

Placement of tNGS is advisable at reference laboratories for optimal utilisations and cost-effectiveness. Laboratories already conducting molecular assays such as LPAs could easily adapt existing spaces for NGS workflows.^5^ At least three well-defined wet laboratory areas are required for 1) sample processing and DNA extraction, 2) preamplification steps, and 3) all amplification/post-amplification procedures. When starting from culture isolates, DNA extraction procedures require BSL3 containment. To realise the full potential of tNGS, samples testing MTB-positive by currently used molecular tests (GeneXpert, TrueNat (Molbio Diagnostics, Goa, India) or TB-LAMP (Eiken Chemical Company Ltd, Tokyo, Japan) can be used, particularly for patients with presumptive DR-TB. Within a network of laboratories implementing NGS, public online NGS forums (like Biostars or SEQ answers^23–25^) among clinicians and laboratorians could help facilitate information exchange, troubleshooting and data interpretation

Study limitations included the inability to access pDST for newer drugs (BDQ) and the optimal TAT for tNGS could not be determined. The prevalence of DR-TB in the Mumbai East-Ward cohort may not accurately reflect the population, as the study underwent a deliberate shift in the selection of RIF-resistant samples over susceptible ones. This was done to gain a better understanding of the mutations currently circulating in the population with regards to second-line drugs.

## CONCLUSIONS

This study demonstrates that reference TB laboratories that already conduct molecular assays in high DR-TB incidence settings could easily adapt existing workstreams for the implementation of tNGS. However, further optimisation of the workflows, including that of the result reporting format, and ongoing training of clinicians on tNGS utility, could improve efficiencies. In high DR-TB settings, tNGS can provide a rapid resistance determination, which can allow earlier initiation of effective treatment, including for newer anti-TB drugs.
